# Supplementary material for: Fast lithium growth and short circuit induced by localized-temperature hotspots in lithium batteries
Source: Nat Commun. 2019 May 6;10:2067. doi: 10.1038/s41467-019-09924-1 (PMC6502817; doi:10.1038/s41467-019-09924-1)
Supplement: Supplementary file 1 — Supplementary Information [file 41467_2019_9924_MOESM1_ESM.pdf]

Supplementary Information

Fast lithium growth and short circuit induced by localized-temperature hotspots in lithium batteries

Yangying Zhu[1#], Jin Xie[2,1#], Allen Pei[1], Bofei Liu[1], Yecun Wu[1,3], Dingchang Lin[1], Jun Li[1,4], Hansen Wang[1], Hao Chen[1], Jinwei Xu[1], Ankun Yang[1], Chun-Lan Wu[1], Hongxia Wang[1], Wei Chen[1], Yi Cui[1,5]*

[1]Department of Materials Science and Engineering, Stanford University, Stanford, CA 94305, USA

[2]School of Physical Science and Technology, ShanghaiTech University, Shanghai 201210, China

[3]Department of Electrical Engineering, Stanford University, Stanford, CA 94305, USA

[4]Department of Chemistry, Stanford University, Stanford, CA 94305, USA

[5]Stanford Institute for Materials and Energy Sciences, SLAC National Accelerator Laboratory, 2575 Sand Hill Road, Menlo Park, CA 94025, USA

[#]These authors contributed equally: Yangying Zhu, Jin Xie

*Corresponding author. Email: yicui@stanford.edu

Supplementary Notes

1. Line heating experiment

To further validate that the morphology change of the deposited Li originated from the local high temperatures and not the photons in the laser, a set of line heating experiments was also conducted. We investigated the same coin cells (CR 2032) as described in Methods. However, instead of creating a hotspot with laser, a thin-film (Pt) resistive line heater (2 mm long, 100 μm wide) was patterned on the backside of the Cu plated thin glass (145 μm thick). A power of 0.03 W was applied to the heater. The temperature distribution was modeled in COMSOL assuming joule heating in the line heater (Supplementary Figure 1a). The result suggests a localized elliptical-shaped hot region with a peak temperature of 54 ºC. The widening of the hot region was mainly due to heat spreading in the 145 μm thick glass which separated the heater and the Cu current collector.

During the experiment, Li was deposited at 1 mA/cm^2 for 2 minutes. The coin cells after Li deposition were then disassembled immediately inside the glove box. The working electrodes were rinsed with DEC to remove salts for SEM imaging. The Li deposition morphology (Supplementary Figure 1b) was carefully examined across the heated region (Supplementary Figure 2c-d: red circle for high temperature; Supplementary Figure 1e-f: orange circle for medium temperature; Supplementary Figure 1g-h: blue circle for low temperature). The results were similar to the uniform temperature experiments, where Li formed at high temperature was less dendritic, and *vice versa*. In addition, Li deposited in the hot region was densely packed, whereas the deposited Li was sparser in the low temperature region. This suggests that Li deposition rate was higher in the high temperature region, which agrees with the hotspot experiment.

2. Uniform temperature experiment

To investigate lithium (Li) deposition morphology at uniform temperature conditions, we tested the same coin cells (CR 2032) as described in Methods in a temperature controlled environmental chamber (BTU-133, ESPEC). The entire coin cell was placed in the environmental chamber for two hours to reach the desired temperature. The same Li deposition current (1 mA/cm^2) was applied for the same period of time (2 minutes) for all the coin cells at different temperatures (room temperature, 30 ºC, 50 ºC, 70 ºC and 90 ºC). The coin cells after Li deposition

were then disassembled immediately inside the glove box. The working electrodes were rinsed with diethyl carbonate (DEC) to remove salts for SEM imaging (Supplementary Figure 2).

The morphology of Li was more dendritic at lower deposition temperature, compared to Li deposited at higher temperature. More specifically, the deposited Li formed whiskers at room temperature. When the temperature increased, the diameter of Li whiskers increased. At 90 ºC, pancake shaped Li with less dendrites was formed.

3. COMSOL simulation – temperature distribution

Model description

The temperature profile as a result of the laser heating was simulated using COMSOL Multiphysics with the "Heat Transfer in Solids" module. A two-dimensional (2D) geometry with rotation was built which forms a three-dimensional (3D) spatial model. The domain consists of a glass disk (145 μm thick, 5.6 mm in radius), a Cu layer (170 nm thick, 5.6 mm in radius) and electrolyte (100 μm thick, 5.6 mm in radius) which represents the experimental conditions. The porous separator which is soaked in the electrolyte domain was not considered in the simulation. This is because it is far from the hotspot, and its thermal conductivity is similar with the electrolyte (both approximately 0.3 W/m·K). The base area is 1 cm^2.

Supplementary Figure 3 shows a zoomed-in view of the three-layer stack. A heat source was created in the Cu by applying uniform volumetric heat generation in a region (50 nm in thickness, 500 nm in radius) to reflect the laser spot size (wavelength of 532 nm, numerical aperture (NA) of 0.6) and the absorption depth (50 nm). The thermo-physical properties of glass, Cu and electrolyte (1 M $LiPF_6$ in 1:1 EC/DEC) is listed in Supplementary Table 2. The absorption of 532 nm laser on Cu is 0.4, [1] and therefore the heating power was 0.4 × the incident laser power.

The governing equation is the Fourier's Law for heat conduction, and stationary conservation of energy.

$$\vec{q} = -k\nabla \mathrm{T}$$

$$\nabla \cdot \vec{q} = Q$$

where T is the temperature, $\vec{q}$ is the heat flux, k is the thermal conductivity, and Q is volumetric heat generation.

The boundary conditions were set as follows: (1) the bottom surface of the electrolyte was fixed at room temperature, since it is in contact with high thermal conductivity Li metal on top of

a thick stainless steel at room temperature; (2) the vertical z axis is axi-symmetric; and (3) the remaining surfaces are adiabatic.

For boundary condition (3), we neglected natural convection and radiation through the top glass surface, since the temperature on the top glass surface is estimated to be near room temperature, and heat transfer coefficient of natural convection of air is also small (~ 10 $W/m^2 \cdot K$). This assumption was validated by additional simulation where the top glass surface has a heat transfer coefficient of 20 $W/m^2 \cdot K$, which resulted in the same hotspot temperature.

Mesh

Due to the various length scale involved (from 50 nm to 5.6 mm) in the COMSOL model, meshing was constructed with very fine meshes near the hotspot to capture the detailed physics, and coarse mesh at a distance from the hotspot to reduce computation time, where it is anticipated that the temperature is basically at room temperature. The meshes (Supplementary Figure 4) were constructed with sizes of (1) 25 nm for the heat source domain (500 nm in radius, 50 nm in thickness); (2) 25 nm to 200 nm for the copper layer; (3) For the glass and electrolyte, the minimum mesh sizes were the same with the hotspot and Cu where the domains connect, and the maximum size was 100 μm (the entire disk is 5.6 mm in radius). To ensure the meshes are fine enough, we reduced the minimum mesh sizes from 25 nm to 10 nm in the heat source domain. The resulting peak temperature was 362.394 K for the original mesh, and 362.395 K for the finer mesh (incident laser energy of 13.4 mW, absorption of 0.4). This suggests that the original mesh is sufficiently fine for the modeling.

Results of the temperature distribution

Results of the temperature distribution with parameters in Supplementary Table 2 are shown in Supplementary Figure 5 (zoomed-in side view) for incident laser powers of 6.7 mW, 13.4 mW and 16.8 mW, respectively. The peak temperature increases with increasing incident laser power.

Supplementary Figure 5 also shows that the temperature on the top Cu surface (i.e., Cu-glass interface) is similar to the temperature on the bottom Cu surface (i.e., Cu-electrolyte interface), since the Cu is thin with high thermal conductivity. This ensures that the graphene temperature indicator can accurately sense the temperature at the Cu-electrolyte interface where

Li is being deposited. More specifically, the peak temperatures on the top and bottom Cu surfaces for the three laser powers are listed in Supplementary Table 1.

In addition, the radial temperature profile along the bottom Cu surface (in contact with the electrolyte) is shown in Supplementary Figure 6a (within 100 μm) and Supplementary Figure 6b (within 1.5 mm).

Dependence on the laser beam profile

Another factor to consider is the shape of the laser beam. In the COMSOL model we approximated the laser beam with a top-hat shape (uniform heating within the laser spot radius σ of 500 nm, and no heating outside the laser spot). For comparison, we also performed simulation using a more detailed Gaussian profile spot. Here the beam radius σ corresponds to the location where the laser power decays to $1/e^2$ of the peak intensity. To result in the same total heating power, the constant A in the Gaussian profile $\rho(r) = Ae^{-\frac{2r^2}{\sigma^2}}$ was obtained by enforcing the total laser power $P_{total} = \int_{r=0}^{r=\infty} Ae^{-\frac{2r^2}{\sigma^2}} 2\pi r dr = A\pi\sigma^2/2$. For implementation of the Gaussian spot in COMSOL numerical model, the Gaussian beam was applied until $r = 2\sigma$ (σ = 500 nm), since at 2σ the beam power $\rho(r = 2\sigma)$ already decayed to a negligible value of 0.03%, and the total power within the circle of $r = 2\sigma$ is 99.966%. Note that here we also used an absorption of 0.4 for the incoming laser energy. The temperature distribution from the Gaussian beam power are shown in Supplementary Figure 7d-f, which is similar to result from the top-hat shape laser profile (Supplementary Figure 7a-c). For convenience, the temperature distribution for the manuscript was based on a top-hat shaped laser spot.

Dependence on thermal conductivity of Cu and electrolyte

We would like to note that the thermal conductivity listed in Supplementary Table 2 for Cu is from reference [2] for thermally evaporated thin-film Cu (thickness approximately 200 nm, grain size of 100 nm), which is lower than the bulk Cu of 385 W/m·K. However, sputtered Cu may have different grains or structures that further lower its thermal conductivity. For reference, we have also calculated the temperature field with a Cu thermal conductivity of 300, 200 and 100 W/m·K, as shown in Supplementary Figure 8 (for incident power of 13.4 mW). The peak temperature increased from 89 ºC (for 350 W/m·K), to 98 ºC (300 W/m·K), 127 ºC (200 W/m·K)

and 201 ºC (for 100 W/m·K). However, it is unlikely that the thermal conductivity of the sputtered Cu in the experimental is so low.

In addition, there lacks available literature data on the thermal conductivity of 1 M $LiPF_6$ in 1:1 EC/DEC electrolyte. Reference [3] suggests a thermal conductivity of 0.45 W/m·K for 1 M LiPF6 in EC/DMC/EMC (not in EC/DEC). Reference [4] measured a thermal conductivity of 0.14-0.16 W/m·K (temperature from 300-350 K) for dimethyl carbonate (DMC), which is chemically similar to DEC. Reference [5,6] suggests thermal conductivity for similar chemical compounds are in the range of 0.1-0.2 W/m·K. Reference [7] listed a thermal conductivity of electrolyte of 0.6 W/m·K, without specifying which electrolyte was used. In light of these references, we used an estimated thermal conductivity of 0.3 W/m·K for 1 M $LiPF_6$ in 1:1 EC/DEC electrolyte in this study (as listed in Supplementary Table 2).

To account for the uncertainty of electrolyte thermal conductivity on the temperature distribution, we calculated the temperature for the glass-Cu-electrolyte system with a range of thermal conductivity for the electrolyte (0.1 W/m·K to 0.6 W/m·K). The result is listed in Supplementary Table 3, where the peak temperature only varies by 3.7%. This indicates the temperature is not sensitive to thermal conductivity of the electrolyte.

Effect of the heat source spot size

In the experiment the focused laser spot size was 1 μm, which was non-adjustable. To investigate the effect of the heat source size, we performed simulation varying the spot size of the heat source, while conserving the total energy input (13.4 mW incident laser with an absorption of 0.4). The radius of the laser spot was varied from 300 nm, 500 nm, 1 μm to 2 μm. The results of the temperature distribution are shown in Supplementary Figure 9a-d, and the radial temperature profile on the Cu-electrolyte interface is shown in Supplementary Figure 9e. Accordingly, lithium deposition current density is also affected, as shown in Supplementary Figure 16.

Heat spreading in the deposited Li

The thermal simulations discussed above was only appropriate for the initial stage (t=0 s), where Li has not formed on the Cu current collector. After Li deposition, the Li layer with higher thermal conductivity than the electrolyte could spread the heat from the laser hotspot and therefore change the temperature distribution. Although the exact model is difficult to construct since the

deposited Li is nanostructured, for an estimation we constructed a simplified model which included a Li disk (1 μm thick, 5 μm in radius) (Supplementary Figure 10). We assumed an effective thermal conductivity of 68 W/m·K for the Li disk (80% lithium (85 W/m·K) and 20% electrolyte (0.3 W/m·K)). The peak temperatures are 47.6, 75.3 and 89.1 °C respectively which are lower than the peak temperatures without the Li disk (55, 90 and 108 °C). In addition, the temperatures on the bottom surface of the Li (or the Li-electrolyte interface) are even lower with a wider width (Supplementary Figure 10). These results suggest that thermal spreading may be one of the reasons that the deposited Li on the hotspot in Figure 2 in the manuscript is much wider than the width of the initial temperature distribution.

4. COMSOL simulation – Lithium deposition rate

The electrochemical simulations were performed using COMSOL Multiphysics with the physics module "Tertiary Current Distribution, Nernst-Planck" and a 2D geometry with rotation, forming a 3D spatial model. The simulation cell (Supplementary Figure 11) consists of a cylinder of electrolyte with base area of 1 cm^2 and height of 100 μm.

For the electrochemical model, governing equations include the following:

Net current density (i_L) within electrolyte is the sum of fluxes of all ions, where F is the Faraday constant, z_i is the charge of the ion, and N_i the flux of species i:

$$i_{ely} = F \sum_i z_i N_i$$

The flux of species i by diffusion and migration (no convection), where D_i, μ_i, and c_i are the diffusion coefficient, mobility, and concentration of species i, respectively, and φ_l is the electrolyte potential:

$$N_i = -D_i \nabla c_i - z_i \mu_i F c_i \nabla \varphi_l$$

Current in the electrolyte is conserved:

$$\nabla \cdot i_{ely} = 0$$

Electrolyte neutrality:

$$\sum_i z_i c_i = 0$$

Overpotential of electrochemical reaction (η), where φ_s is the electrode potential and E_{eq} is the equilibrium potential of the Li/Li^+ couple.

$$\eta = \varphi_s - \varphi_l - E_{eq}$$

Butler-Volmer equation, which governs the local current density, with j_0 being the exchange current density, α_a and α_c being the anodic and cathodic charge transfer coefficient, respectively, R, the gas constant, and T, the temperature:

$$j = j_0 \left(e^{\frac{\alpha_a F \eta}{RT}} - e^{\frac{-\alpha_c F \eta}{RT}} \right)$$

The boundary conditions and initial conditions are as follows. The diffusion coefficient of Li^+ in the electrolyte was set to 3.2×10^{-6} cm^2/s from reference[8] and initial Li ion concentration was set to 1 M. One end of the electrolyte cylinder was set as the counter electrode, acting as a lithium source with current density 1 mA/cm^2. The other end of the cylinder was the Cu working electrode where Li deposition occurs. The electrode potential at the reaction interface is held at 0 V.

To generate local heating at the Cu working electrode, the temperature profile results from the laser heating simulation were interpolated into the electrochemical model. The temperature profile was used to scale the exchange current density for the Li/Li^+ electrochemical reaction by the following Arrhenius relation:

$i_0 = \exp[(-E_a/R)*1/T(r) + 32.01]$

where E_a is the activation energy, calculated from experiments as 73.5 kJ/mol, R is the gas constant, T(r) is the temperature profile in Kelvin resulting from the laser heating as a function of r, the radius from the center of the laser spot, and 32.01 is a constant that results from the data fitting. The experiments for calculating the activation energy consisted of measuring the Li/Li^+ exchange current density at different temperatures using a microelectrode, following the methods in Shi et al.[9]. The slope of the linear fit of the plot of 1000/T vs. $\ln(j_0)$ gives the activation energy (Supplementary Figure 12).

In this model, we have only considered the scaling of the exchange current density (through Arrhenius scaling of the rate constant) with temperature and neglected the effects of the enhancements of ionic conductivity of the electrolyte. We found that even when implementing the highest laser power of 16.7 mW and with the resulting maximum local current density of 311.2 mA/cm^2 at the laser spot center, there was minimal changes in the concentration within the electrolyte (*i.e.* no depletion of Li^+ at the laser spot). The concentration at the center of the laser spot was still 0.9 M at steady state (Supplementary Figure 13), as compared to the initial

concentration of 1 M. If the electrolyte conductivity was significantly enhanced, for example by local heating of the electrolyte from the laser spot, even the most extreme case of having no concentration gradient would result in a maximum concentration of 1 M at the laser spot center. Based on concentration-dependent Butler Volmer kinetics (where current scales linearly with changes in concentration gradient), this would only increase the current density by around 11%, or around a 1.1 times increase (green line in Supplementary Figure 14). In contrast, the increases in current density from the scaling of the rate constant/exchange current density are significantly greater due to the exponential nature of the Arrhenius relation (blue line in Supplementary Figure S14).

The time-dependent solution was solved until steady state was reached (i.e., current density and concentration profiles were steady). Variations in deposition rate (current density) at the working electrode resulting from local heating were observed. The result of the Li current density along the radial r direction is shown in Supplementary Figure 15. Similar to the temperature profiles, the Li current density also decays sharply with r. In addition, since the temperature distribution depends on the laser spot size, lithium deposition current density is also laser spot size dependent, as shown in Supplementary Figure 16.

5. Optical cell hotspot temperature simulation

To estimate the temperature of the hotspot in the optical cell (Supplementary Figure 17a), we constructed a 3D model in COMSOL which includes a 12 μm thick Cu current collector (k of 385 W/m·K for bulk Cu) surrounded in electrolytes, and a heat source of 1 μm in diameter and 50 nm in thickness at the top edge of the Cu. The heat transfer coefficient was 100 W/m^2K at the electrolyte-Cu surface, and temperature of the electrolyte at a distance to Cu was at room temperature. The temperature distribution near the hotspot is shown in Supplementary Figure 17b, for an incident laser power of 13.4 mW. The peak temperature is approximately 43 ºC.

Supplementary Figures

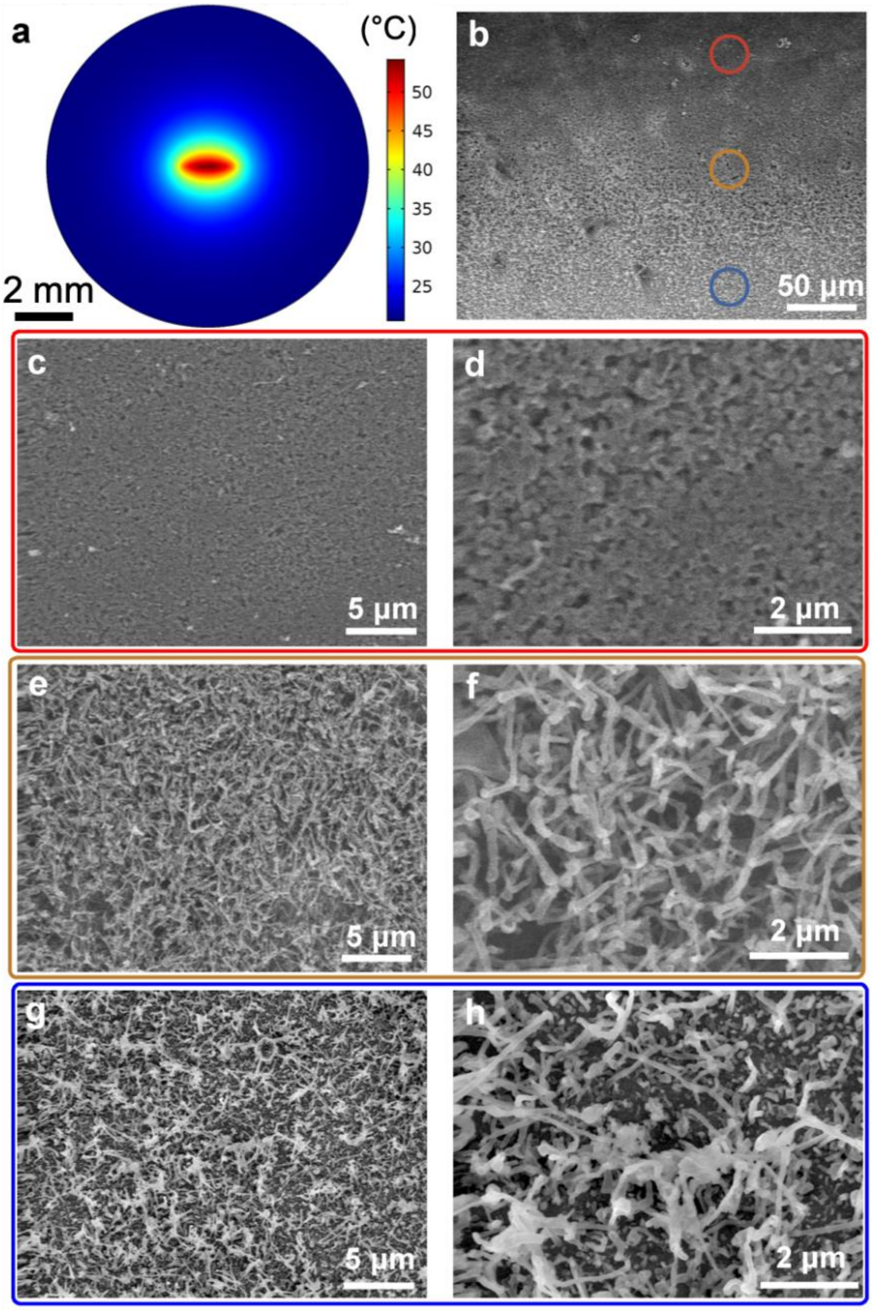

Supplementary Figure 1. COMSOL simulation and SEM characterization of Li deposited on a line-shaped hot region. (a) Temperature distribution (simulated in COMSOL) on the Cu/glass current collector as a result of heating from a thin-film line heater on the backside of the glass. (b) Low magnification SEM image of Li deposition on a line heater. SEM images of Li deposited in the (c-d) high temperature region, (e-f) medium temperature region, and (g-h) low temperature region.

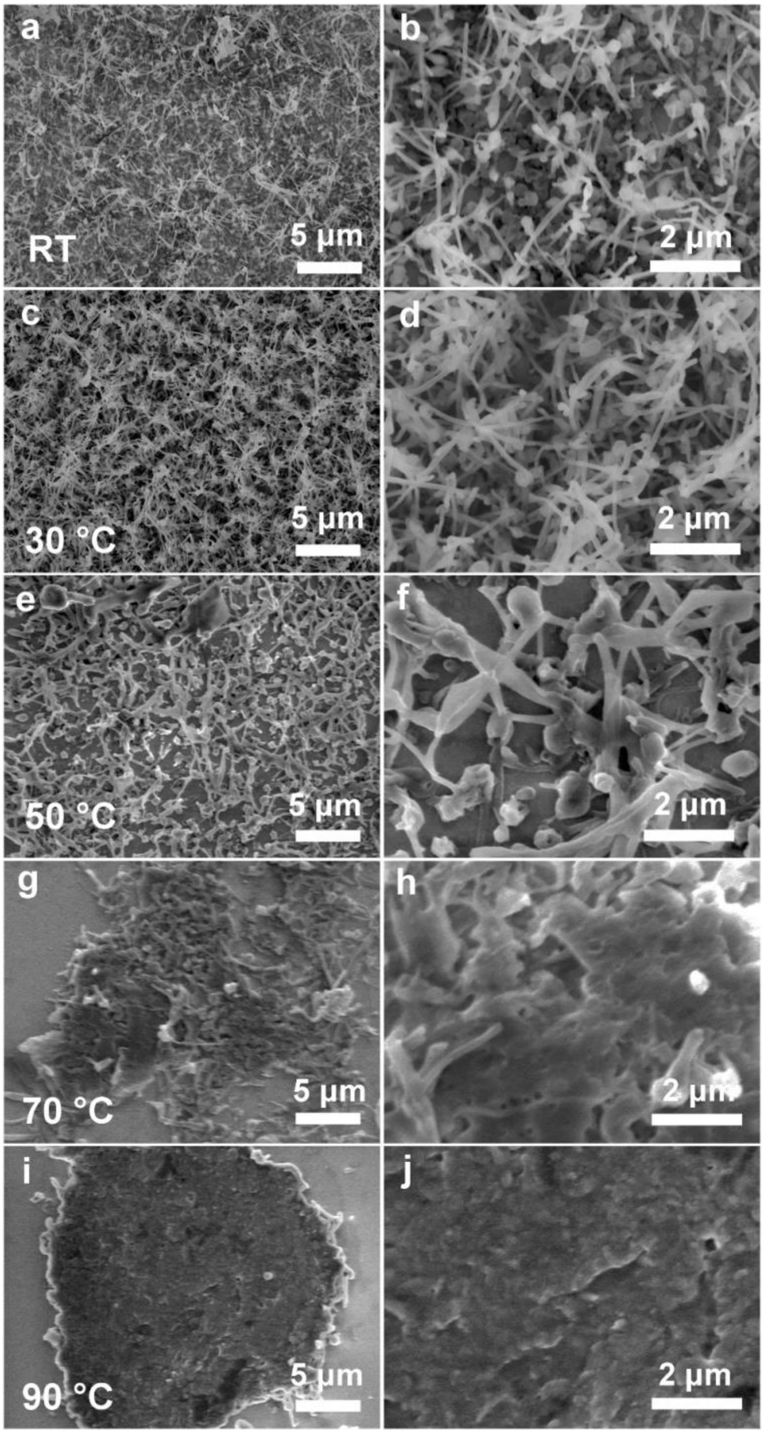

Supplementary Figure 2. SEM characterization. SEM images of Li plated on Cu electrodes at (a-b) room temperature (RT); (c-d) 30 ºC; (e-f) 50 ºC; (g-h) 70 ºC; and (i-j) 90 ºC.

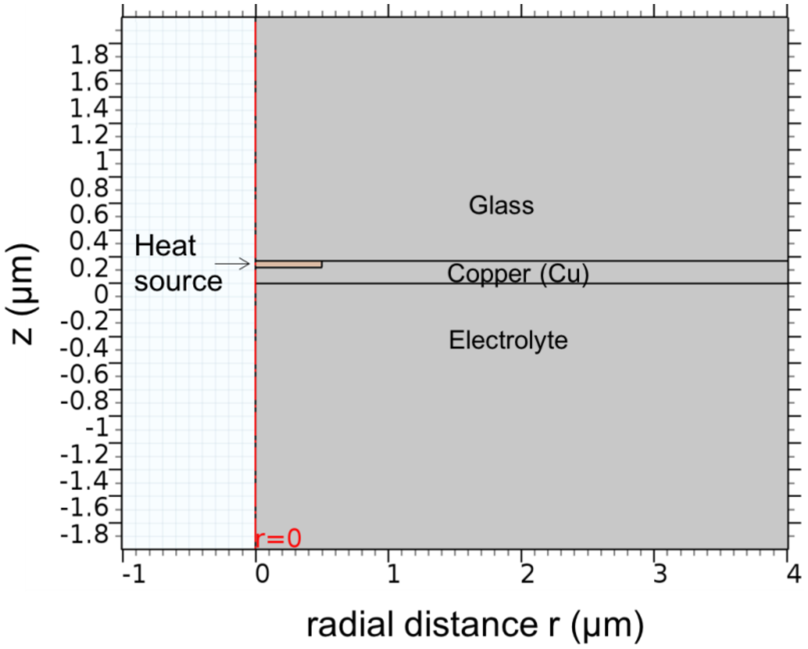

Supplementary Figure 3. Thermal model. Zoomed-in view of the thermal simulation cell which consists of a top glass layer (145 µm thick, 5.6 mm in radius), a Cu layer (170 nm thick, 5.6 mm in radius), electrolyte (100 µm thick, 5.6 mm in radius), and a heat source in the Cu layer. The entire geometry is not shown due to the much larger length scale compared to the heat source.

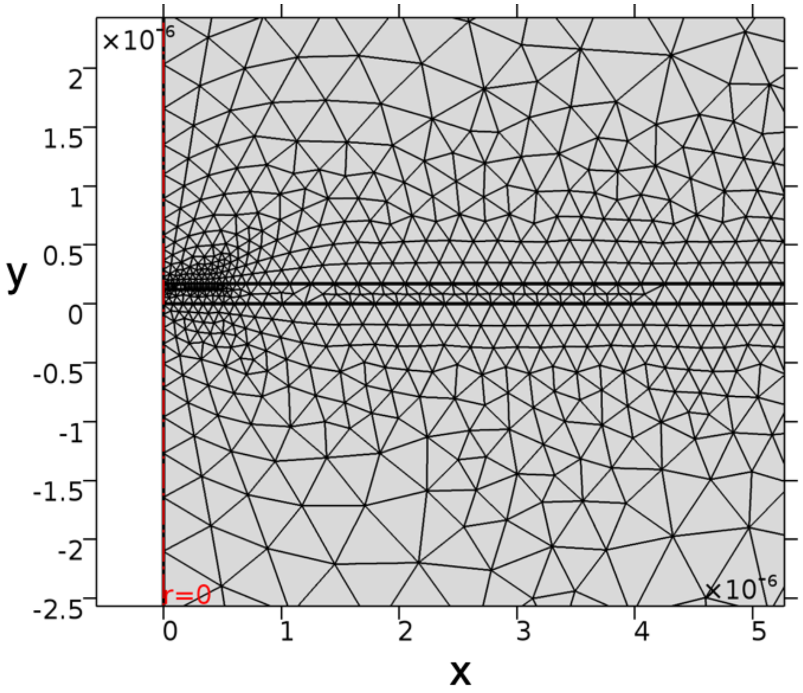

Supplementary Figure 4. Mesh near the laser spot.

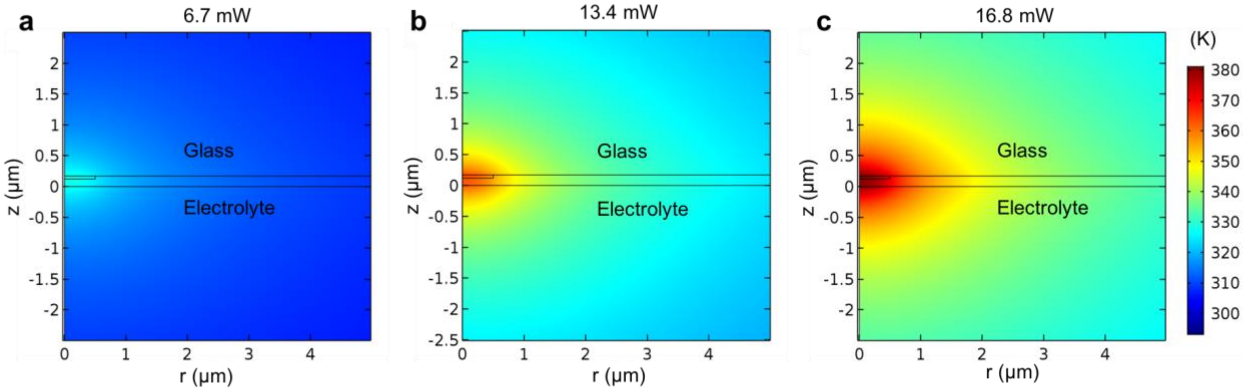

Supplementary Figure 5. Temperature distribution (zoomed-in side view) for incident laser powers of (a) 6.7 mW, (b) 13.4 mW and (c) 16.8 mW, respectively. The top layer is glass, the middle layer is Cu, and the bottom layer is electrolyte.

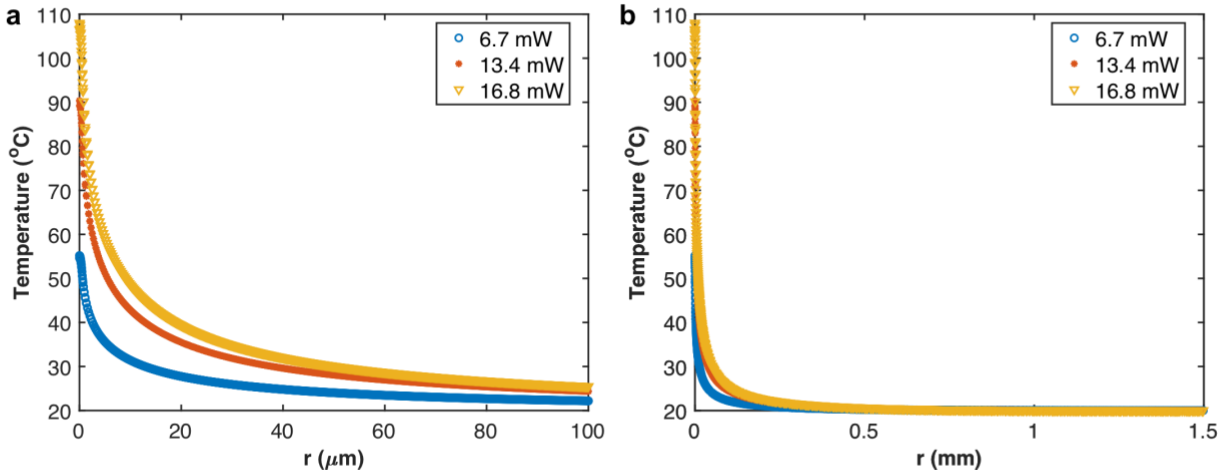

Supplementary Figure 6. The radial temperature profile along the bottom Cu surface for incident laser powers of 6.7 mW, 13.4 mW and 16.8 mW, respectively. (a) r up to100 μm and (b) r up to 1.5 mm.

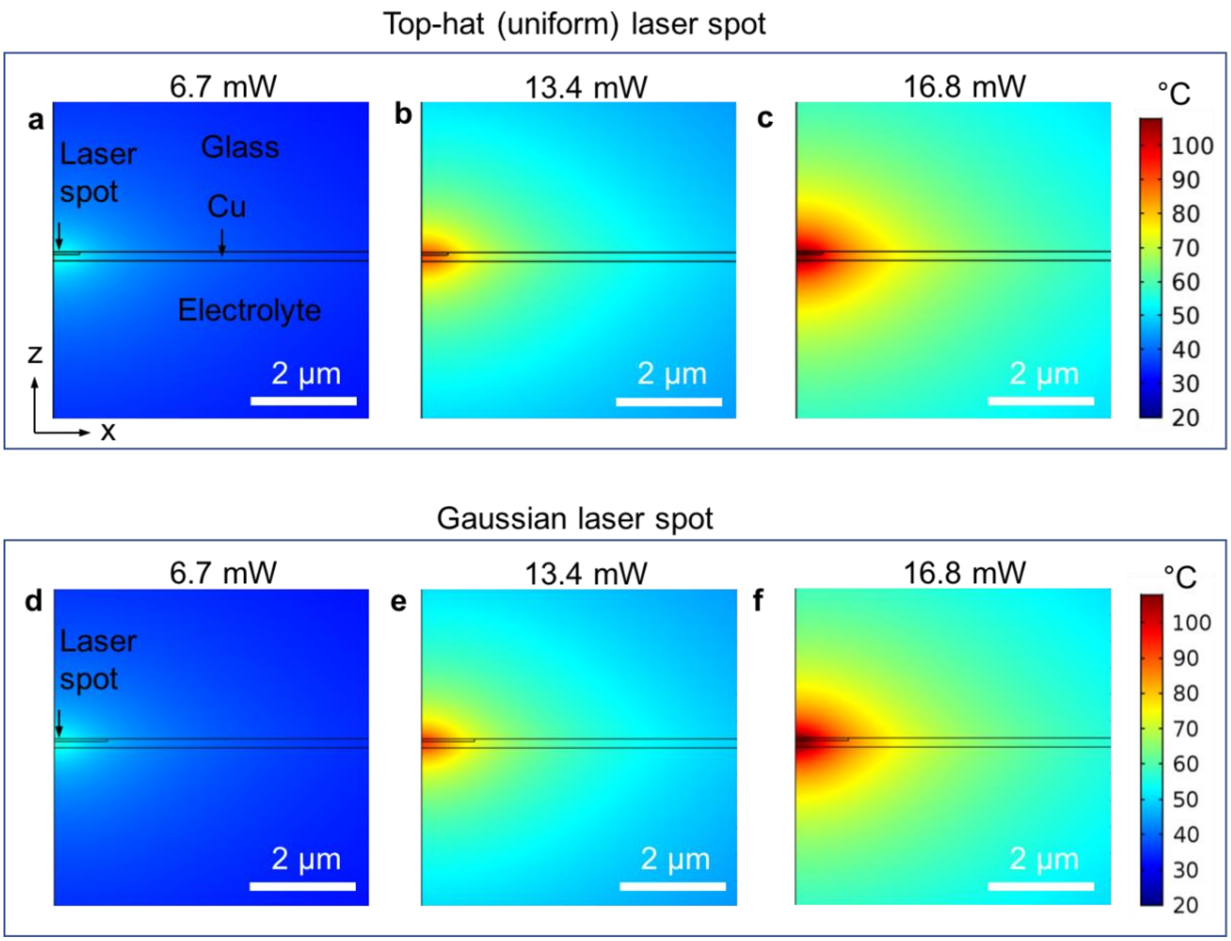

Supplementary Figure 7. Temperature distribution (zoomed-in side view) for (a-c) a top-hat shaped laser spot with uniform power within a radius of 500 nm, and (d-f) a Gaussian shaped laser with a same radius of 500 nm (at which location the Gaussian intensity decays to $1/e^2$ of the peak value), and the Gaussian beam was applied until r = 1 um. The temperature distributions were similar between the two laser patterns.

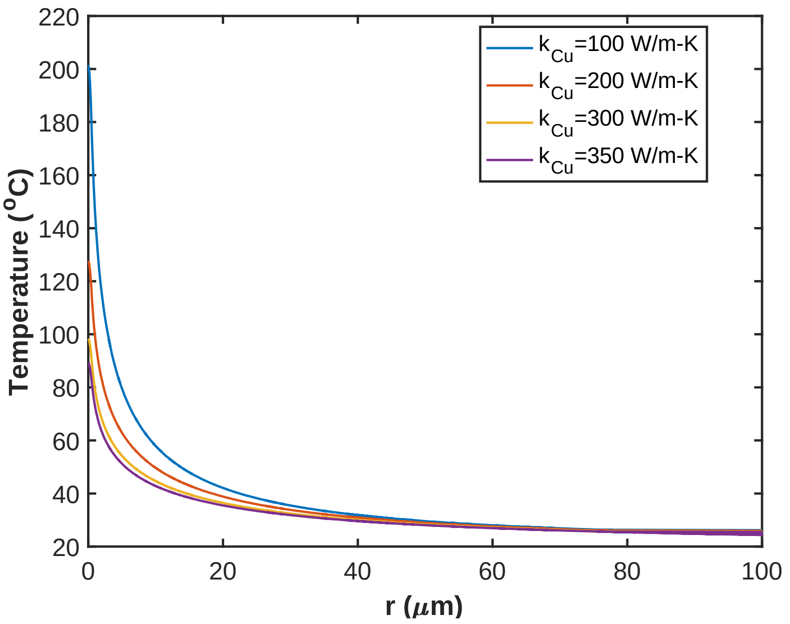

Supplementary Figure 8. The radial temperature profile along the bottom Cu surface for incident laser power of 13.4 mW, with the thermal conductivity of the Cu layer varying from 350 W/m·K (used in this work) to 100 W/m·K.

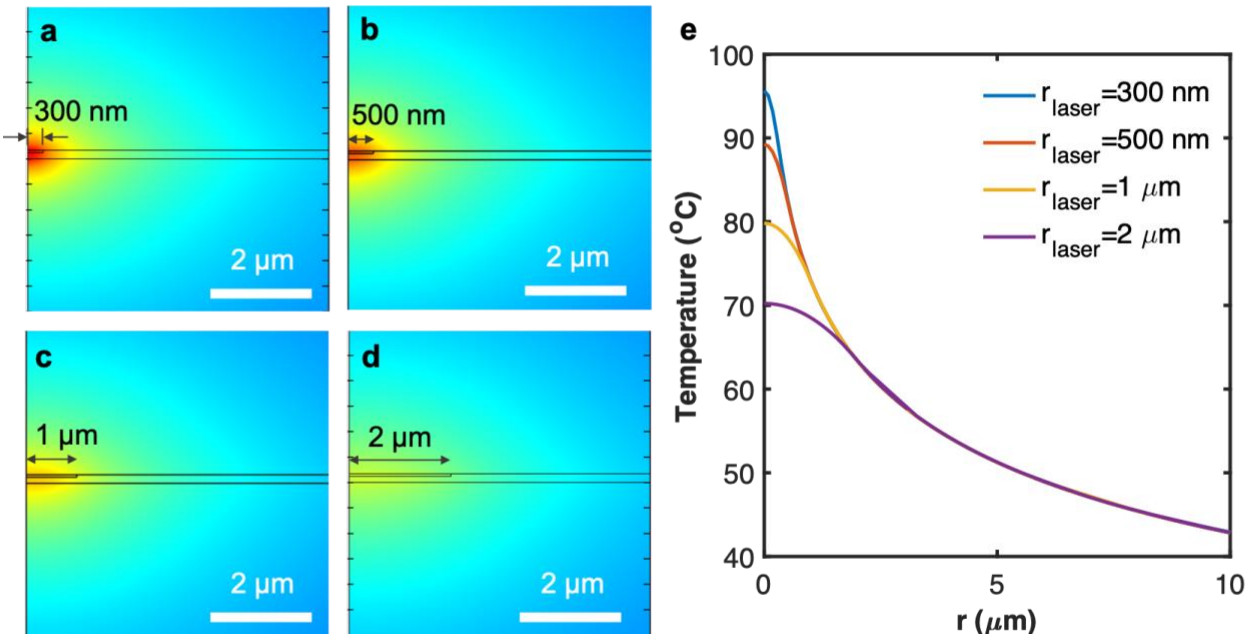

Supplementary Figure 9. Temperature distribution (zoomed-in side view) with a laser spot radius of (a) 300 nm, (b) 500 nm, (c) 1 μm and (d) 2 μm. The incident laser power is 13.4 mW (532 nm, absorption of 0.4). The 500 nm radius beam (diameter of 1 μm) was the actual laser beam size used in the experiment. (e) Radial temperature profile on the Cu-electrolyte interface.

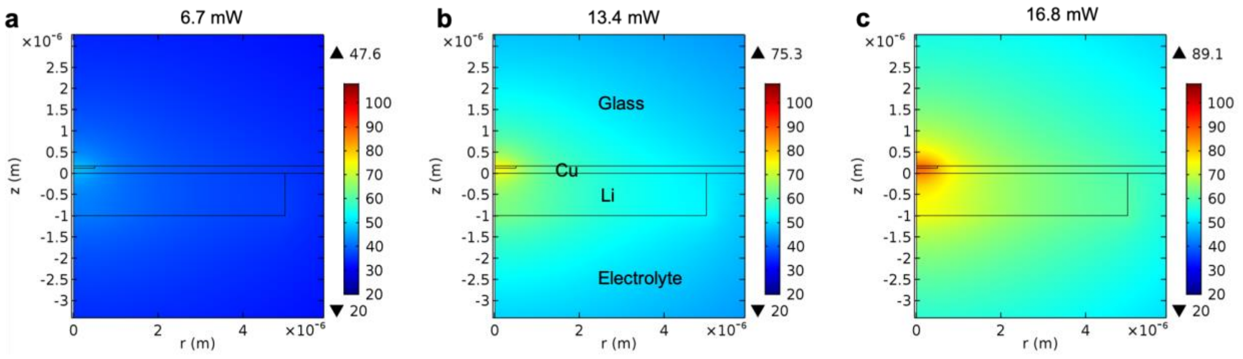

Supplementary Figure 10. Temperature distribution (zoomed-in side view) near a uniform laser spot with a Li-disk (1 μm thick, 5 μm in radius) on the Cu current collector. The incident laser powers are (a) 6.7 mW, (b) 13.4 mW and (c) 16.8 mW, respectively.

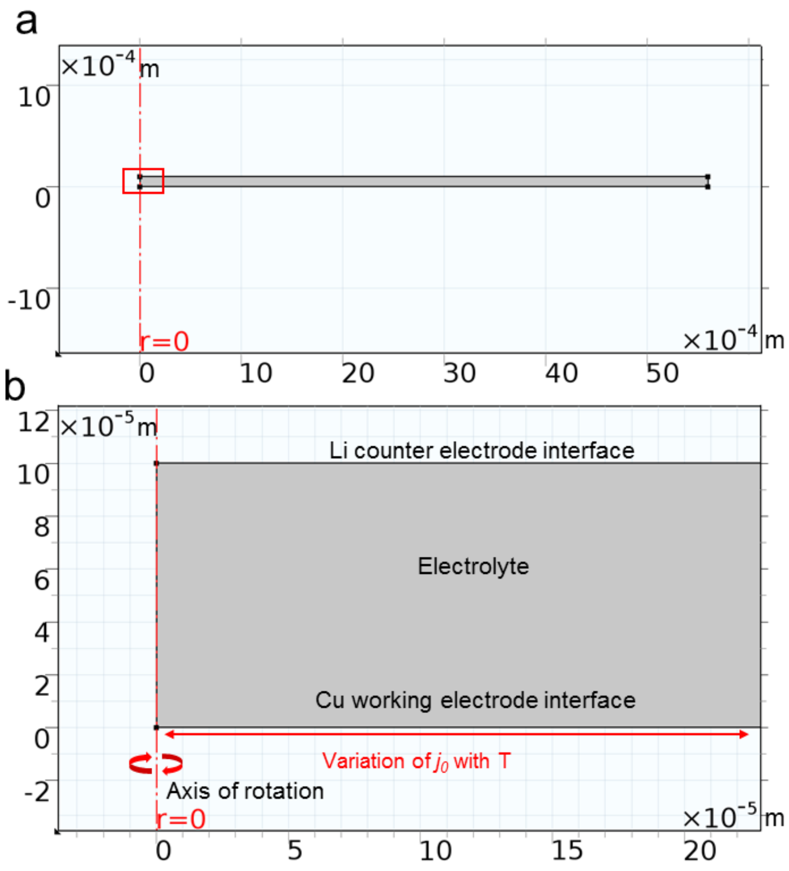

Supplementary Figure 11. (a) Overall geometry of the simulation cell. The overall electrode area is 1 cm^2 after rotation around the r = 0 axis. (b) Zoomed-in image on the red box in (a),

indicating the electrode interfaces and electrolyte. The variation in exchange current density is applied at the working electrode interface, along the red arrow over the entire electrode surface.

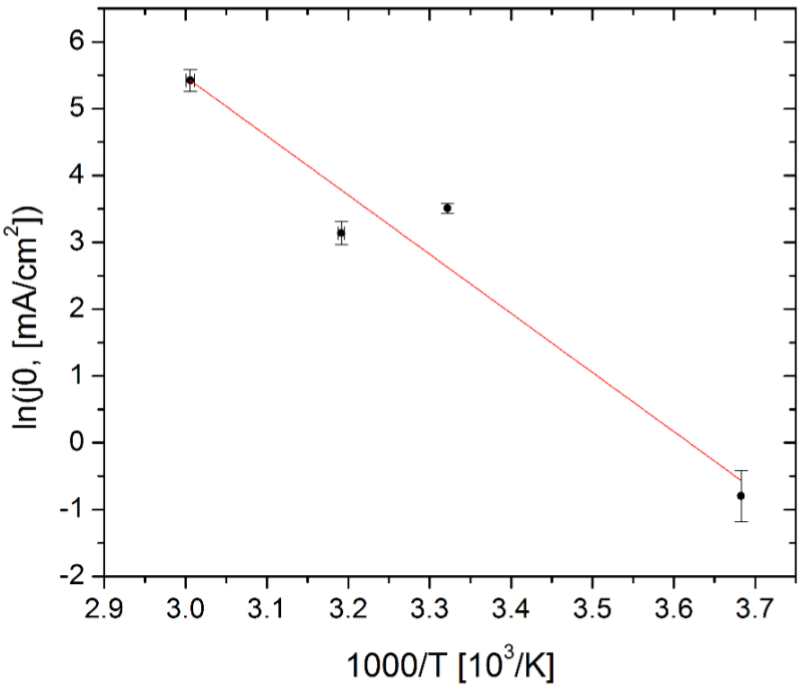

Supplementary Figure 12. Arrhenius plot of exchange current density measured using microelectrode at different temperatures for the Li/Li^+ electrochemical reaction at the Li metal surface.

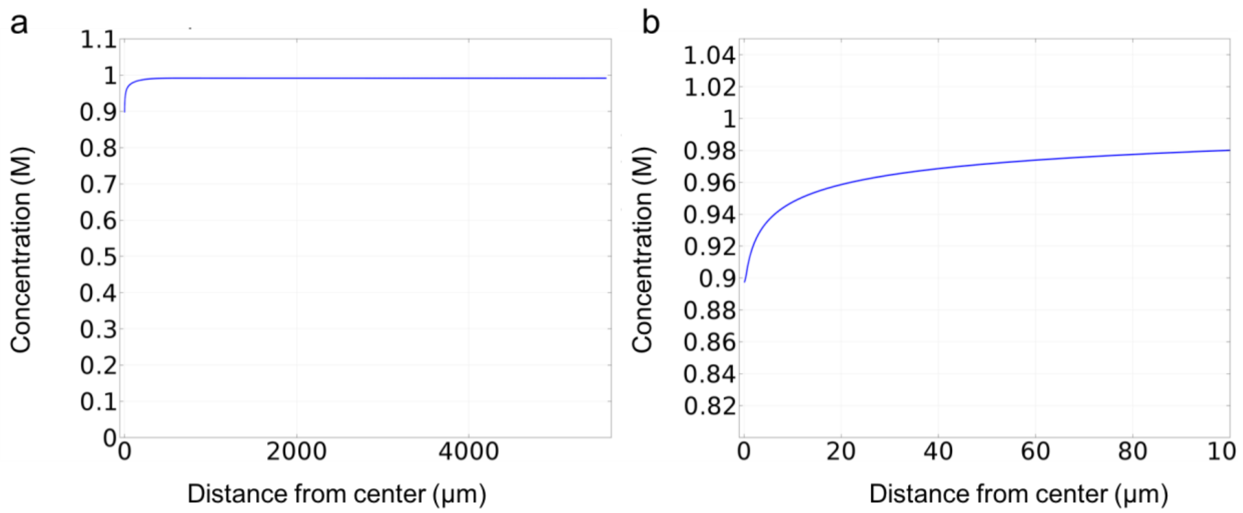

Supplementary Figure 13. (a) Plot of concentration at the depositing electrode interface at steady-state over the entire cell. Initial concentration is 1 M. (b) Zoomed concentration plot within 100

microns of the laser spot. The minimum concentration is at the center of the laser spot where the local current density is highest, with a minimum value of 0.9 M.

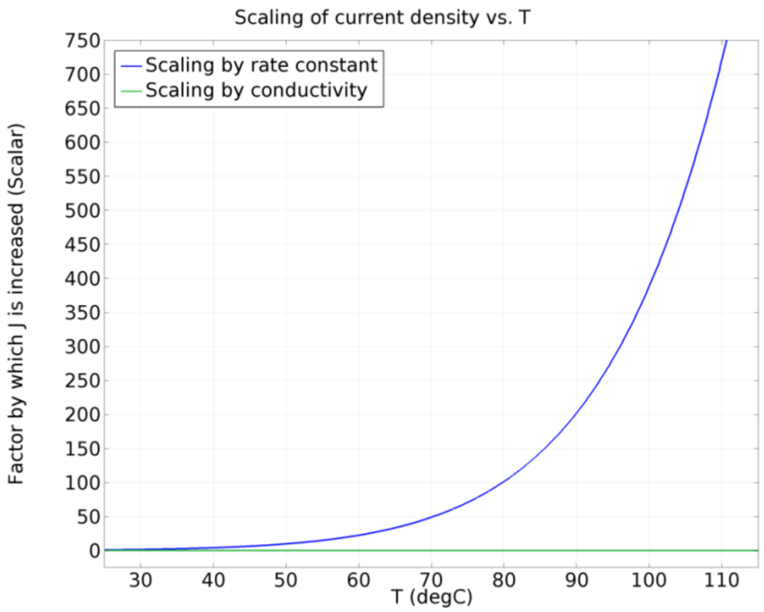

Supplementary Figure 14. Scaling of exchange current density with temperature due to changes in the rate constant and conductivity of the electrolyte.

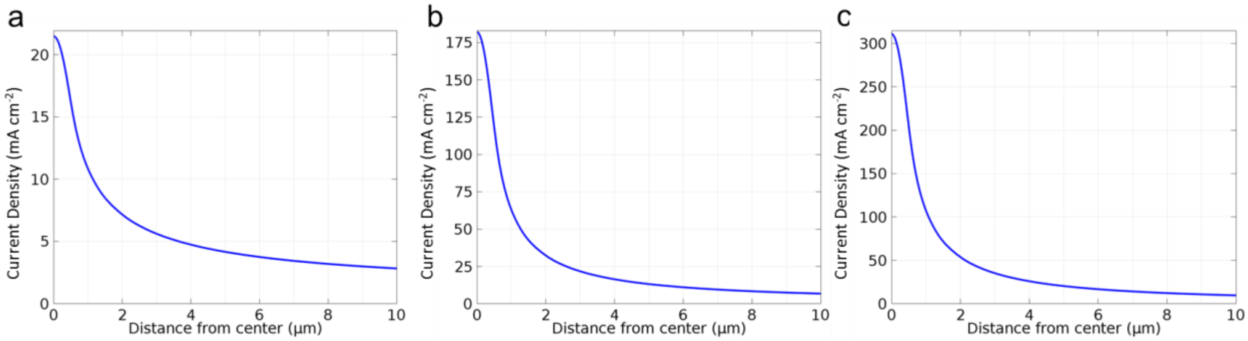

Supplementary Figure 15. Local current density at the electrode interface at distances away from the laser spot center for incident laser powers of (a) 6.7 mW, (b) 13.4 mW, and (c) 16.8 mW.

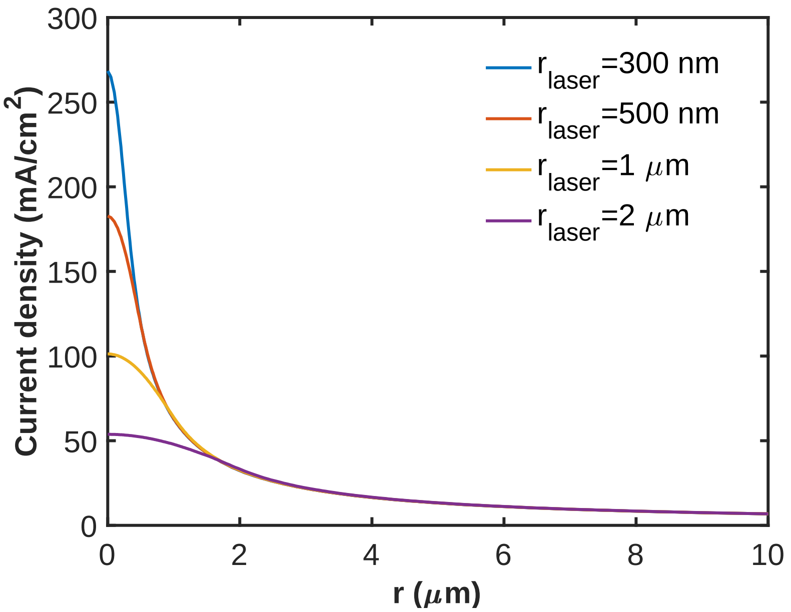

Supplementary Figure 16. Li-deposition current density radial distribution for a laser spot radius of 300 nm, 500 nm, 1 μm and 2 μm. The incident laser power is 13.4 mW (532 nm, absorption of 0.4).

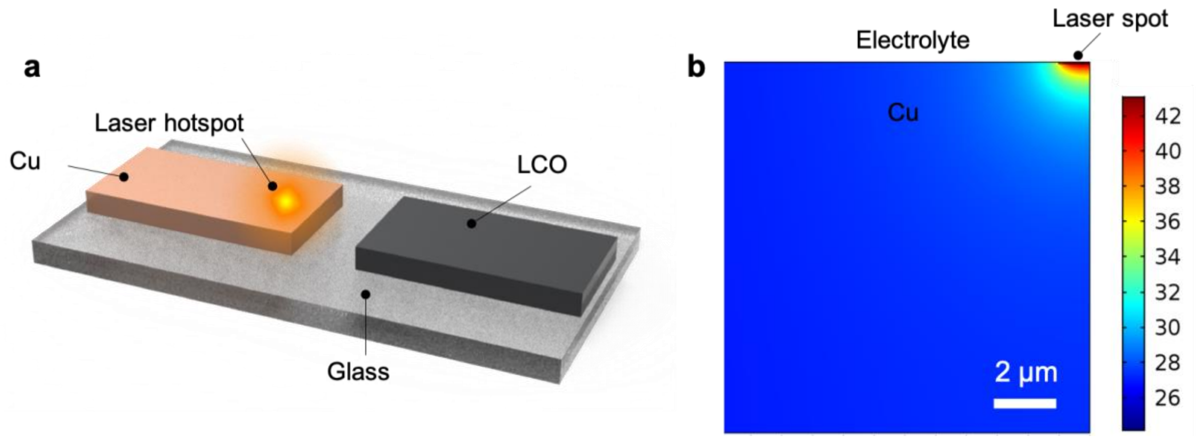

Supplementary Figure 17. (a) Schematic of the optical cell. (b) Temperature distribution in the Cu foil. The laser was focused on the top right corner. The hotspot temperature is approximately 43 ºC.

Supplementary Tables

Supplementary Table 1. Simulated peak temperature on both sides of the Cu current collector.

Laser power	Peak temperature at the Cu-glass interface	Peak temperature at the Cu-electrolyte interface
6.7 mW	55.2 ºC	54.6 ºC
13.4 mW	90.4 ºC	89.2 ºC
16.8 mW	108.0 ºC	106.6 ºC

Supplementary Table 2. Thermo-physical properties of glass, Cu and electrolyte (1 M $LiPF_6$ in 1:1 EC/DEC) used in the COMSOL model.

Property	Glass	Cu	Electrolyte
Heat capacity C_p (J/kg·K)	703	384	1778
Density (kg/m^3)	2203	8960	1260
Thermal conductivity (W/m·K)	1.38	350*	0.3**

* estimated from reference [2], also discussed later in this section
** estimated from reference [3–7], also discussed later in this section

Supplementary Table 3. Peak temperature for various thermal conductivity of the electrolyte.

Thermal conductivity of the electrolyte (W/m·K)	Maximum temperature (ºC) from simulation
0.1	93.7
0.2	91.8
0.3 (used in the manuscript)	90.4 (used in the manuscript)
0.4	89.3
0.5	88.3
0.6	87.5

Supplementary References:

1. Johnson, P. B. & Christy, R. W. Optical constants of the noble metals. *Phys. Rev. B* **6**, 4370–4379 (1972).
2. Lugo, J. M. & Oliva, A. I. Thermal Diffusivity and Thermal Conductivity of Copper Thin Films at Ambient Conditions. *Journal of Thermophysics and Heat Transfer* **30**, 481–489 (2016).
3. Guo, G. *et al.* Three-dimensional thermal finite element modeling of lithium-ion battery in thermal abuse application. *Journal of Power Sources* **195**, 2393–2398 (2010).
4. Jin, X., Wu, J., Liu, Z. & Pan, J. The thermal conductivity of dimethyl carbonate in the liquid phase. *Fluid Phase Equilibria* **220**, 37–40 (2004).
5. Yaws, C. L. *Handbook of Thermal Conductivity, Volume 1: Organic Compounds C1 to C4*. (Gulf Professional Publishing, 1995).
6. Yaws, C. L. *Handbook of Thermal Conductivity, Volume 2: Organic Compounds C5 to C7*. (Elsevier, 1995).
7. Chen, S. C., Wan, C. C. & Wang, Y. Y. Thermal analysis of lithium-ion batteries. *Journal of Power Sources* **140**, 111–124 (2005).
8. Valøen, L. O. & Reimers, J. N. Transport properties of LiPF6-based Li-ion battery electrolytes. *J. Electrochem. Soc.* **152**, A882–A891 (2005).
9. Shi, F. *et al.* Strong texturing of lithium metal in batteries. *PNAS* **114**, 12138–12143 (2017).
